# Clinico-pathologic profile of skin cancers in oculocutaneous albinism at Universitas Academic Hospital

**DOI:** 10.4102/hsag.v30i0.2906

**Published:** 2025-04-23

**Authors:** Molikuoa Harriet Makuru, Frans Maruma, Edward Ngwenya, Kelvin Mponda

**Affiliations:** 1 Department of Dermatology, Faculty of Health Sciences, University of the Free State, Bloemfontein, South Africa; 2 Department of Plastic and Reconstructive Surgery, Faculty of Health sciences, University of Pretoria, Pretoria, South Africa; 3 Department of Dermatology, Queen Elizabeth Central Hospital, Blantyre, Malawi

**Keywords:** oculocutaneous albinism (OCA), skin cancer, SCC (squamous cell carcinoma), BCC (basal cell carcinoma), melanoma

## Abstract

**Background:**

Oculocutaneous albinism (OCA) is a genetic disorder found worldwide. Skin cancer is a significant risk for people with albinism, particularly in Africa, where it is a major cause of death. Many patients delay seeking medical care until their skin lesions are in advanced stages.

**Aim:**

The aim of this study was to describe the clinico-pathological profile of skin cancers in patients with albinism at their initial presentation to our dermatology outpatient department.

**Setting:**

This study was conducted at the dermatology department of Universitas Academic Hospital, Bloemfontein, South Africa.

**Methods:**

A retrospective descriptive study covering June 2009 to July 2019 was conducted. Only records of oculo-cutaneous albinism patients diagnosed with skin cancer during their initial visit were included.

**Results:**

Eighty-six patients with albinism were recorded, 37% (*n* = 32) of whom had skin cancer at their first visit. Females (81%) were more affected than males (19%). The majority of skin cancers were squamous cell carcinomas (SCCs) (54%) and basal cell carcinomas (BCCs) (46%). No melanomas were found. Most SCCs were classified as aggressive (80.4%), compared to 30.8% of BCCs.

**Conclusion:**

Almost 40% of OCA patients presented with skin cancer at their initial visit, highlighting the need for strengthening primary healthcare systems’ efficiency in ensuring early referrals for OCA patients.

**Contribution:**

Education, socioeconomic support and awareness campaigns are *sine qua non* actionable factors that could help encourage early medical evaluation for all OCA patients.

## Introduction

Albinism is a genetically inherited disorder with a worldwide distribution (Kiprono, Chaula & Beltraminelli 2014). Phenotypically, it presents as reduced or absent melanin in the hair, skin and eyes (Kiprono et al. 2014). There are two main types of albinism: ocular albinism (OA), which affects only the eyes, and oculocutaneous albinism (OCA), which affects both the eyes and the skin (Mabula et al. 2012). Genetically, albinism is classified into eight types based on specific gene mutations (eds. Bolognia, Schaffer & Cerroni 2018; Lekalakala et al. 2015; Pennamen et al. 2021). Among these, oculocutaneous albinism type 2 (OCA2) is the most common form worldwide (eds. Bolognia et al. 2018; Fajuyigbe et al. 2018).

Skin cancer is the most common malignancy among Caucasians but is generally rare in black Africans. However, OCA is an established risk factor for skin cancer in the black African population (Kiprono et al. 2014; Lekalakala et al. 2015). The lack of melanin, combined with increased ultraviolet (UV) radiation exposure, significantly heightens the risk of skin cancer in individuals with albinism (Kiprono et al. 2014). In OCA, melanin production predominantly involves pheomelanin (yellow or red melanin), with minimal eumelanin (brown or black melanin) production (eds. Bolognia et al. 2018; Fajuyigbe et al. 2018). Eumelanin provides effective photoprotection, while pheomelanin offers limited protection against UV radiation and generates carcinogenic reactive oxygen species (ROS) during its biosynthesis (eds. Bolognia et al. 2018; Fajuyigbe et al. 2018; Lekalakala et al. 2015). The reduced photoprotection caused by the lack of eumelanin, combined with ROS generation from pheomelanin, contributes to the development of keratinocyte cancers (Enechukwu et al. 2020; Fajuyigbe et al. 2018; Lekalakala et al. 2015). Consequently, patients with OCA have a significantly higher risk of developing skin cancer than the general black African population.

Squamous cell carcinoma (SCC) is the most common skin cancer diagnosed in OCA, accounting for 75% – 88% of cases, followed by basal cell carcinoma (BCC) (9% – 23%) and rare cases of melanoma (1.3% – 3%) (Enechukwu et al. 2020; Binesh, Akhavan & Navabii 2010; Mabula et al. 2012). Skin cancer is a significant complication of albinism and a leading cause of death in Africans with albinism (Mabula et al. 2012). It is estimated that 98% of people with albinism die before the age of 40, with skin cancers accounting for 80% of these deaths (Enechukwu et al. 2020; Awe, Abudu & Oyelekan 2015; Binesh et al. 2010; Lekalakala et al. 2015). This high mortality rate is often attributed to delays in seeking or receiving medical treatment for premalignant or malignant actinic lesions (Awe et al. 2015; Kiprono et al. 2014).

The aim of this study was to describe the clinico-pathological profile of skin cancers in patients with albinism at their initial presentation to our dermatology outpatient department. In addition, the study sought to determine whether these cancers were infiltrating (aggressive) or superficial (non-aggressive), thereby providing valuable insights for raising awareness about cancer prevention among both patients and healthcare providers.

## Research methods and design

### Study design

This was a retrospective descriptive study covering a 10-year period from July 2009 to June 2019.

### Setting

The study was conducted in the Dermatology Outpatient Department of Universitas Academic Hospital in Bloemfontein, South Africa. This tertiary hospital is academically affiliated with the University of the Free State. Notably, it is the only state facility providing dermatological care and services in the entire Free State province.

### Aim and objectives

1. To determine the prevalence of albinism patients in the outpatient database
2. To assess the prevalence of skin cancers among OCA patients in the dermatology outpatient clinic’s database during their first visit
3. To describe the clinical and histopathological characteristics of those with skin cancers.

### Study population and sampling strategy

A non-probability convenience sampling method was used to access clinical records of patients diagnosed with OCA and reviewed in accordance with ethical guidelines. The inclusion criteria consisted of patients diagnosed with skin cancer at their initial presentation to our centre. Patients who had been diagnosed before referral to our clinic, as well as those diagnosed at subsequent visits, were excluded.

### Data collection

A standardised data sheet was used to collect variables, including age, gender, comorbidities, premalignant lesions, clinical and histological descriptions of malignant lesions, and the date of first presentation at our dermatology clinic. The anatomical site of the lesions and treatments administered or performed was not within the scope of this study.

**FIGURE 1:**
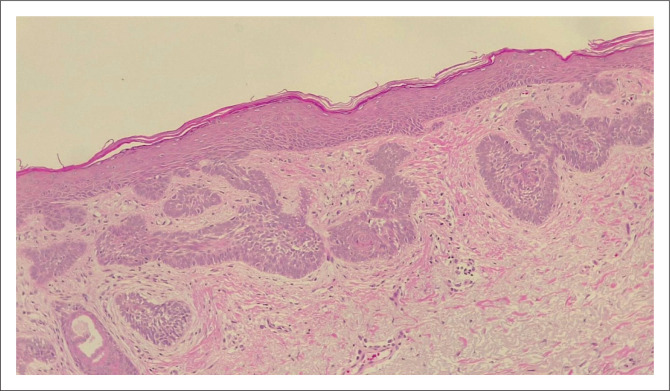
(×10 Magnification) Superficial spreading basal cell carcinoma (non-aggressive).

**FIGURE 2:**
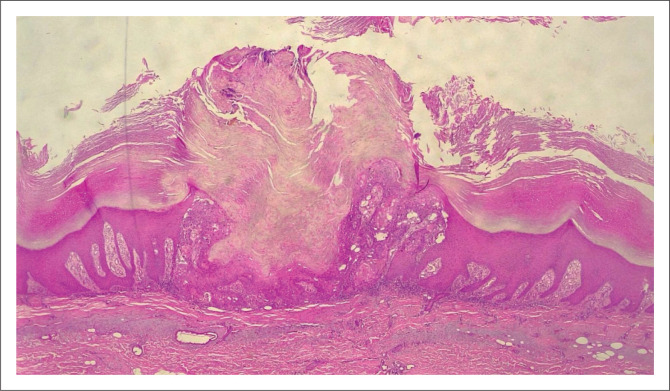
(10× Magnification) Squamous cell carcinoma *in situ* (non-aggressive).

**FIGURE 3:**
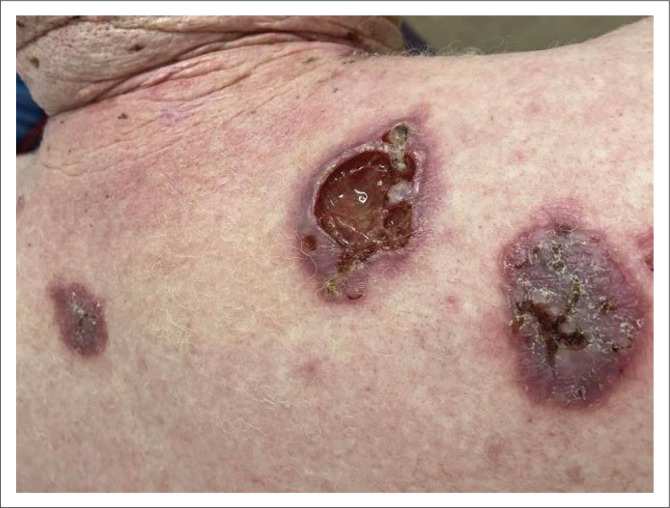
Ulcerated lesion of aggressive basal cell carcinoma (middle lesion), superficial spreading squamous cell carcinoma (right), and a satellite small superficial spreading basal cell carcinoma (left) appearing on the upper back of the same patient.

**FIGURE 4:**
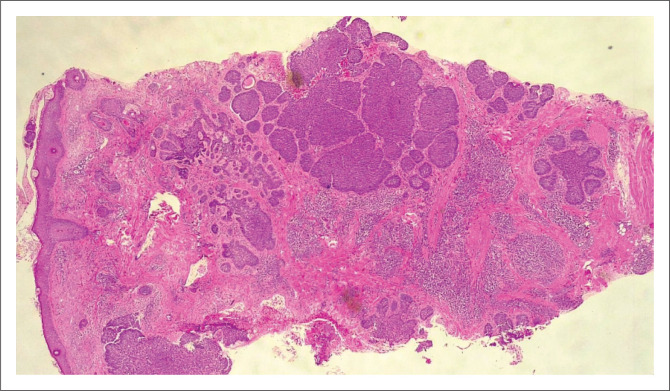
(4× Magnification) Infiltrating basal cell carcinoma extending into deeper structures (aggressive).

**FIGURE 5:**
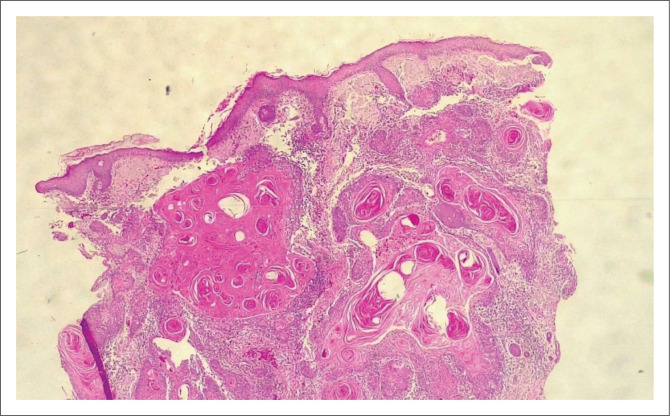
(4× Magnification) Infiltrating squamous cell carcinoma that is moderately differentiated (aggressive).

**FIGURE 6:**
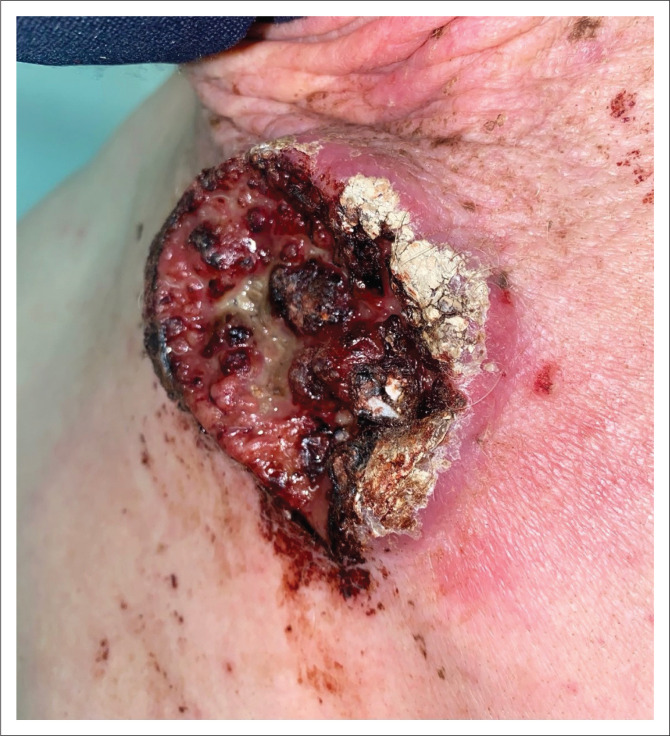
Aggressive ulcerated squamous cell carcinoma upon superior xiphisternum.

The clinical morphology of the lesions was recorded as nodules, plaques, ulcers or other (papules and patches). Histological changes were categorised as aggressive or non-aggressive. The authors defined the aggression of skin cancers as follows:

- *Non-aggressive* (NA) cancers are limited to the layers of the epidermis (horizontal growth pattern) and are referred to as superficial and non-infiltrating (Figure 1, Figure 2 and Figure 3).
- *Aggressive* (A) cancers exhibit ulceration, a vertical growth pattern, and invasion of subcutaneous tissue and underlying structures, that is, infiltrating cancers (Figure 4, Figure 5 and Figure 6) for photomicrographs and images of typical lesions from the cohort’s archives).

### Data analysis

Data were encoded using MS Excel, and Stata MP version 14 software was used for data processing and analysis. Continuous variables were presented as means with standard deviations (s.d.) or medians with interquartile ranges (IQR), depending on the data distribution. Categorical variables were presented as frequencies and percentages.

### Ethical considerations

Ethical clearance was granted by the Health Sciences Research Ethics Committee, Faculty of Health Sciences, University of the Free State (reference no: UFS-HSD2019/2232/2403). Further approval was obtained from the Free State Provincial Health Department. The histological photomicrographs used in this article were generated by the researchers themselves from the histopathological slides and clinical pictures obtained from the departmental archives after the gate keeper’s permission. Written informed consent was obtained from all individual participants involved in the study.

## Results

A total of 86 (*N* = 86) patients with OCA were documented at our clinic from July 2009 to June 2019. Out of these, 35 (*n* = 35) patients met the initial inclusion criteria. However, three participants were excluded due to insufficient data, resulting in a final study population of 32 patients (*n* = 32). The mean age of the study population was 42.97 years, with ages ranging from 16 to 69 years. The mean age at which these patients were diagnosed with skin cancer was 48 years. The total lesion count of skin cancers was 85 in the cohort included for analysis (*n* = 32). Table 1 provides further details in this regard.

**TABLE 1:** Descriptive statistics for continuous variables illustrating the range, means, standard deviations and the interquartile ranges.

Variables	*n* = 32			Range
	Mean ± s.d.	Median	[IQR]	
Age	42.97 ± 13.96	-	-	16–69
**Morphologic variables**				
No. of SCC lesions (*n* = 46)	-	1	1–2	1–7
No. of BCC lesions (*n* = 39)	-	2	1–3.5	1–5
No. of melanoma lesions (*n* = 0)	-	-	-	-

s.d., standard deviation; IQR, interquartile range; SCC, squamous cell carcinoma; BCC, basal cell carcinoma; No., number.

In terms of comorbidities, 3.1% (*n* = 1) had type II diabetes mellitus. Hypertension was documented in 15.6% (*n* = 5) of the patients while 18.5% (*n* = 6) of the patients were documented as HIV and AIDS positive.

### Types of skin cancers detected

Squamous cell carcinomas was the most common histological type of cancer detected in 54.1% (*n* = 46) (followed by BCC 45.9% (*n* = 39) of the patients. The median number of lesions was four for SCC and 3 for BCC. Forty per cent (*n* = 13) of patients were detected with both SCC and BCC lesions simultaneously; however, no case of melanoma lesion was detected. Regarding the extent of cancer spread, none of the patients had metastasis recorded. It was beyond this study’s objectives to determine the number of cancers per patient, albeit such information would be interesting. Over 80% (*n* = 37) of SCC lesions were classified as aggressive, compared to just over 30% (*n* = 12) of BCC lesions. Table 2 provides further details in this regard.

**TABLE 2:** Clinical morphology and aggressiveness of the lesions on histology.

Clinical morphologic feature	Cancer type			
	SCC (*n* = 46)		BCC (*n* = 39)	
	*n*	%	*n*	%
Nodules	16	34.8	10	25.6
Plaques	15	32.6	14	35.9
Ulcers	12	26.1	15	38.5
Other (papules and patches)	3	6.5	0	0.0
**Aggressiveness**				
Aggressive	37	80.4	12	30.8
Non-aggressive	9	19.6	27	69.2

SCC, squamous cell carcinoma; BCC, basal cell carcinoma.

### Dermatoheliosis

Evidence of sun damage as represented by the presence of solar keratosis, actinic telangiectasia, cutis rhomboidalis nuchae, solar lentigines was recorded in all 32 patients (100%) evaluated in this study.

Table 2 illustrates the clinical aspects as well as the histopathological features of the skin cancers recorded in this cohort.

## Discussion

Persons with albinism in Africa are more prone to early cutaneous sun damage, which often results in the early onset of skin cancers. This is likely because of the region’s higher levels of ultraviolet radiation and climatic conditions. Previous studies have reported that skin cancers related to albinism typically develop at a mean age of below 40 years (Kiprono et al. 2014; Mabula et al. 2012). However, in this study, the mean age of diagnosis for skin cancers was 48 years. Consistent with trends in the literature, no melanomas were observed in this study, with all cases being non-melanoma skin cancers (NMSC). This aligns with the common finding that NMSCs are the most prevalent histological subtype in persons with albinism, while melanoma remains rare (Kiprono et al. 2014; Mabula et al. 2012; Ruiz-Sanchez, Garabito & Valtueña 2020; Van der Westhuizen et al. 2015). In the analysis of the National Cancer Registry for melanoma in South Africa, Tod et al. (2019) reported a higher incidence of melanoma among Caucasians compared to African blacks, with no cases of melanoma reported among persons with albinism. Some authors have suggested that the low incidence of melanoma in individuals with OCA may be because of a complex interplay between ultraviolet radiation exposure and genetic factors (Kiprono et al. 2014).

This study revealed a higher prevalence of skin cancers in females compared to males which contrasts with other reports. For instance, Enechukwu et al. (2020) found that more males were diagnosed with skin cancer than females in Tanzania. Similarly, Kiprono et al. (2014) and Opara and Jiburum (2010) observed a comparable number of cases in males and females. The gender disparity in this study could be attributed to several factors. According to Andrees et al. (2024), one possible explanation is that women may be more likely to seek medical care, especially when they notice suspicious lesions. However, it remains elusive to determine the exact factors that can explain these findings, given the significant confounding factors such as the small sample size. Therefore, further studies to better understand the epidemiology of OCA in South Africa are still needed.

In line with studies by Kiprono et al. (2014), Mabula et al. (2012), and Lekalakala et al. (2015), the most common cutaneous malignancy in this study was SCC, followed by BCC.

The mean duration of skin lesions before seeking medical help was unclear in most records, with only five patients reporting a mean duration of three years for both BCC and SCC.

Consistent with Andrees et al.s’ (2024) findings, there was, in general, a notable delay in seeking medical attention. Ideally, these patients should be evaluated in early childhood to receive care that could prevent cancers and provide social support to counter the myths and societal beliefs that put them at risk. Delays may be because of a lack of awareness regarding the increased risk of skin cancer in individuals with albinism or socioeconomic factors. Another contributing factor could be the absence of clear policy guidelines instructing healthcare workers to refer all individuals with albinism for dermatological and ophthalmological evaluations, regardless of their initial reason for seeking medical attention. Implementing such guidelines could lead to earlier referrals, potentially reducing morbidity, mortality and disease progression in these patients.

Given the comorbidity profile of this cohort-hypertension, diabetes mellitus and HIV/AIDS, the authors deduce that there was access to healthcare facilities for non-OCA related concerns. When these patients attended clinics for the treatment of their comorbidities, referrals to dermatology should have been made, provided the healthcare providers were informed about the holistic care and needs of individuals with OCA. Similar studies have observed late presentations in other regions as well (Awe & Azeke 2018; Lekalakala et al. 2015; Mabula et al. 2012; Opara & Jiburum 2010).

Persons with albinism often face marginalisation and social discrimination in Southern Africa because of their distinctive appearance among the predominantly dark-skinned population (Brocco 2016; Jones, Smith & Ochieng 2018; Mouhari-Toure et al. 2021; Mswela 2018; Phatoli, Bila & Ross 2015). This may also contribute to delays in seeking medical attention because of fears of rejection. In South Africa, there have been unsuccessful calls to introduce a special disability grant that must be given to all patients with OCA in recognition of the genetic nature of the disease as well as the socioeconomic challenges faced by this population. Although sacrosanct, the eye care, skin care, sun protection and regular specialist visits (dermatologists and ophthalmologists) are quite costly for these patients (Nöthling-Slabbert & Mswela 2013). A more deliberate and intentional effort needs to be made by all stakeholders in achieving this objective, which would improve the lives of persons living with albinism pursuant to the United Nations’ declaration (Mswela 2018).

### Limitations

Given the relatively small sample size of our cohort, further studies are necessary to better define the epidemiology of skin cancers in OCA patients in South Africa. More extensive research will also help to explore the underlying factors contributing to gender disparities, delayed presentations and the low incidence of melanoma in this population. Addressing these challenges through public health education, policy improvements, and further research is critical for reducing morbidity and mortality among persons with albinism.

## Conclusion

This study highlights several important findings related to the incidence and characteristics of skin cancers in persons with albinism in South Africa. Almost 40% of patients presented with skin cancer at their initial visit, highlighting the need for strengthening primary healthcare systems and early referrals for OCA patients. Education, socioeconomic support and awareness campaigns are essential to encourage earlier medical evaluation in attempt to curb the cancer morbidity observed in these patients. The need for an urgent call for a universal disability grant for all OCA patients cannot be over-emphasised.

While previous research has reported the mean age of skin cancer onset below 40 years, this study found a slightly higher mean age (for diagnosis) of 48 years suggesting a late presentation to the dermatology facilities. Consistent with existing literature, the authors observed no melanomas, with all cases being NMSC, specifically SCC’s and BCC’s, with SCC being more aggressive of these subtypes.
